# Genome analysis of a plasmid-bearing myxobacterim *Myxococcus* sp. strain MxC21 with salt-tolerant property

**DOI:** 10.3389/fmicb.2023.1250602

**Published:** 2023-09-18

**Authors:** Lin Liu, Fengjuan Xu, Jinhui Lei, Peiwen Wang, Lei Zhang, Jihong Wang, Jingya Zhao, Dongmei Mao, Xianfeng Ye, Yan Huang, Gang Hu, Zhongli Cui, Zhoukun Li

**Affiliations:** ^1^Key Laboratory of Agricultural Environmental Microbiology, Ministry of Agriculture and Rural Affairs, College of Life Sciences, Nanjing Agricultural University, Nanjing, China; ^2^College of Life Sciences, Nanjing Agricultural University, Nanjing, China

**Keywords:** myxobacteria, salt tolerance, social behavior, adaptive evolution, predation

## Abstract

Myxobacteria are widely distributed in various habitats of soil and oceanic sediment. However, it is unclear whether soil-dwelling myxobacteria tolerate a saline environment. In this study, a salt-tolerant myxobacterium *Myxococcus* sp. strain MxC21 was isolated from forest soil with NaCl tolerance >2% concentration. Under 1% salt-contained condition, strain MxC21 could kill and consume bacteria prey and exhibited complex social behaviors such as S-motility, biofilm, and fruiting body formation but adopted an asocial living pattern with the presence of 1.5% NaCl. To investigate the genomic basis of stress tolerance, the complete genome of MxC21 was sequenced and analyzed. Strain MxC21 consists of a circular chromosome with a total length of 9.13 Mbp and a circular plasmid of 64.3 kb. Comparative genomic analysis revealed that the genomes of strain MxC21 and *M. xanthus* DK1622 share high genome synteny, while no endogenous plasmid was found in DK1622. Further analysis showed that approximately 21% of its coding genes from the genome of strain MxC21 are predominantly associated with signal transduction, transcriptional regulation, and protein folding involved in diverse niche adaptation such as salt tolerance, which enables social behavior such as gliding motility, sporulation, and predation. Meantime, a high number of genes are also found to be involved in defense against oxidative stress and production of antimicrobial compounds. All of these functional genes may be responsible for the potential salt-toleration. Otherwise, strain MxC21 is the second reported myxobacteria containing indigenous plasmid, while only a small proportion of genes was specific to the circular plasmid of strain MxC21, and most of them were annotated as hypothetical proteins, which may have a direct relationship with the habitat adaptation of strain MxC21 under saline environment. This study provides an inspiration of the adaptive evolution of salt-tolerant myxobacterium and facilitates a potential application in the improvement of saline soil in future.

## 1. Introduction

Myxobacteria are considered unique gram-negative bacteria for their social behavior and complex developmental stages. Up to now, myxobacteria, such as *Myxococcus xanthus*, have emerged as the pre-eminent model system to understand the genetics and molecular mechanisms involved in social behaviors (Kaiser, 2003). As micropredator, myxobacteria are able to prey on bacteria and fungi and oomycetes in a possible wolf-pack-like manner (Zhang et al., 2023a), while there is accumulating evidence for predatory mechanisms promoting “selfish” behavior during predation as myxobacteria reduce the transport of lytic factors away from the secretor by increasing the size of cell populations without cell cooperation (Berleman et al., 2016). During this process, lytic enzymes, chemical metabolites, and outer membrane vesicles (OMVs) are deduced to be involved in prey killing (Li et al., 2019; Thiery and Kaimer, 2020; Zhang et al., 2023a). Otherwise, myxobacteria are regarded as microbial factories for production of active secondary metabolites and enzymes (Kaur et al., 2018; Li et al., 2022). These characteristics could allow myxobacteria to survive in different condition, which may also find great application in agriculture and biomedicine and environmental protection.

Myxobacteria are globally distributed and prefer non-saline soil and sediments, followed by saline environments, plant rhizospheres and surfaces, and animal secretions, but also minorly appear in host-associated environments (Wang et al., 2021b). Recently, *Myxococcota* species also show predominant roles in activated sludge (Zhang et al., 2023b). This wide distribution enables myxobacteria to play a major role in determining community structures and potential mass circulation (Wang et al., 2021a). However, limited natural species of myxobacteria are explored. It is a common perception that chances for the discovery of resource novelty increase by moving toward rarely screened myxobacteria, such as underexplored species occupying extraordinary habitats or specific ecological niches (Hoffmann et al., 2018). In contrast to isolates from terrestrial soil, saline habitat represents an extreme environment (Zhang et al., 2020), and halophilic or halotolerant myxobacteria from saline habitat are classified as a special myxobacterial group due to their growth characteristics and sociological behavior under saline condition (Zhang et al., 2005).

Up to now, salt-tolerant myxobacteria are mainly isolated from marine samples, such as seawater and sediments, and marine *Myxococcus fulvus* HW-1 has been regarded as a model strain to investigate the adaptation of social behavior of myxobacteria from soil to oceanic conditions (Pan et al., 2010; Li et al., 2011). Investigation of the salt-tolerant mechanism showed that halotolerant myxobacteria are the result of the degenerative adaptation of soil myxobacteria to the marine environment (Zhang et al., 2005), and their salt tolerance is often closely associated with genes of S-motility, two-component system, and outer membrane component (Pan et al., 2009, 2010). The social behavior of marine salt-tolerant myxobacteria under hypersaline environment represents potential evolutional lineage. Compared with marine enviroment, myxobacterial strains from saline–alkaline soil also have been identified with salt tolerance (Zhang et al., 2013), whereas whether the salt-tolerant myxobacteria from terrestrial system adopt the similar adaptation behavior of marine myxobacteria, and similar machanisms are deployed in response to salt remain to be determined.

In the present study, a halotolerant myxobacterium *Myxococcus* sp. strain MxC21 with favorable NaCl tolerance was isolated from forest soil. Strain MxC21 displayed typical social behaviors with the presence of 1% NaCl and also harbored the ability to prey on bacteria. Previous research studies indicated that adenylyl cyclase and Clp/HSP100 family of molecular chaperones (Kimura et al., 2002; Pan et al., 2012), sodium-hydrogen exchange family protein (Bhardwaj et al., 2018), S-motility-related gene locus (Zhang et al., 2007), and two-component system gene Tc105 (Pan et al., 2010) are involved in salt tolerance of myxobacteria, whereas genome-based information is unexplored. To characterize and explore the potential feathers of salt-tolerant strain MxC21, we assembled the chromosome-level genome of strain MxC21, performed a comparative analysis of genomes and functional genes, and highlighted the different metabolic and signal pathways potentially contributing to salt tolerance.

## 2. Materials and methods

### 2.1. Myxobacteria isolation and culture condition

The model *M. xanthus* DK1622 was cultured on CTT (1% Casitone, 10 mM Tris-HCl; 0.1 mM PBS; and 8 mM MgSO_4_, pH 7.6). *Myxococcus* sp. strain MxC21 (GDMCC No:63059) was isolated from saline soil using the rabbit fecal separation method and purified by repeated inoculation onto VY/4 medium (1% yeast cells and 1% CaCl_2_, w/v, pH 7.0). The obtained isolates were inoculated in LB liquid medium (1% tryptone, 0.5% yeast extract, 0.5% NaCl, and 1% w/v) and cultured overnight at 30°C at 180 rpm. The strain that could grow normally under certain salt concentration was screened and stored at −80°C in glycerol tubes at a final concentration of 16% for further use.

### 2.2. Isolation and identification of candidate myxobacteria with salt tolerance

Approximately 200 g of soil from Xinjiang province of China was collected from the upper 5–15 cm layer. The samples were air-dried as quickly as possible after collection and stored at room temperature. The myxobacteria isolation with induction of fruiting body formation was performed as described before (Li et al., 2018). In brief, fruiting bodies induced on sterile rabbit dung pellets were transferred to VY/4 medium and incubated at 30°C. The emergence of diffuse colonies was observed after 4–7 days of incubation. The strain from the colony edge was transferred to a new medium, and the procedure was repeated until a pure culture was obtained.

For the phylogenetic analysis, the 16S rRNA gene was amplified using the forward primer 20F (5′-AGAGTTTGATCCTGGCTCAG-3′) and the reverse primer 1500R (5′-GGTACCTTGTTACGACTT-3′). The similarity search of the 16S rRNA gene was performed using BLAST from the NCBI database. Multiple alignments of the sequenced nucleotides were generated, and a neighbor-joining tree was constructed using ClustalX (version 2.0) (Thompson et al., 1997) and MEGA 11 (Kumar et al., 2016).

### 2.3. Salt tolerance of myxobacterium MxC21

*Myxococcus* sp. strain MxC21 was cultured in CTT medium at 32°C with the addition of salt when necessary. The effect of sodium chloride (NaCl) on the growth of MxC21 was investigated on CTT agar media with the presence of 0–3% NaCl. Strain MxC21 was cultured on the center of the agar plate and incubated at 30°C for 2 days. Otherwise, strain MxC21 was cultured in CTT liquid medium with 0–2% NaCl at 30°C for 24 h. Due to the clump growth of wild-type myxobacteria in liquid condition, the biomass of strain MxC21 was determined by qRT-PCR. For qPCR, the total DNA of strains from the liquid condition was isolated by liquid nitrogen treatment (AceQ^®^ Universal SYBR qPCR Master Mix, Vazyme Biotech Co., Ltd, China), following the manufacturer's instructions.

### 2.4. Fruiting body formation and motility assays

Fruiting body formation of strain MxC21 was performed on TPM agar plates (10 mM Tris-HCl, pH 7.6, 1 mM KH_2_PO_4_, 8 mM MgSO_4_, and 1.5% Agar), as described before (Skotnicka et al., 2016). In brief, 5 μl of spots containing 5 × 10^9^ cells/ml of strain MxC21 each were placed on TPM agar plates and allowed to develop at 30 °C for 7 days. The fruiting body formation was observed by the formation of aggregates with a stereo microscope (Nikon SMZ1270). Otherwise, 2 μl of spot containing 5 × 10^9^ cells/ml strain MxC21 was spotted onto the CTT plate containing 0.3% agar and 1.5% agar with the presence of 0–1.5% NaCl. The plates were incubated at 32°C for approximately 2–3 days. A- and S-motility were analyzed by observing the colonies for expansion *via* a Nikon SMZ1500 stereo microscope.

### 2.5. Submerge cultural assay

Cells in CTT liquid medium at the mid-exponential phase were washed with ice-cold MOPS buffer (10 mM 3-(N-morpholino)-propanesulfonic acid, 1 mM CaCl_2_, 1 mM MgCl_2_, and pH6.8). The washed cells were resuspended to a final concentration of 5 × 10^9^ cells/ml, and 2 ml of samples were transferred to the 24-well plates with the presence of 0–1.5% NaCl, followed by incubation at 32°C for 4–6 days without shaking, and the aggregations from different treatments were observed (Mironenko et al., 2020).

### 2.6. Predation assays

After activated growth on an agar plate at 30°C for 2 days, strain MxC21 was cultured in 50 ml CTT liquid medium at 30°C for 16 h. *Escherichia coli* was grown in an LB medium to an OD600 of 1.0. Then, the bacterial cells were collected by centrifugation at 10 000 × g for 3 min and washed with TPM buffer three times (10 mM Tris-HCl, pH 7.6, 1 mM KH_2_PO_4_, and 8 mM MgSO_4_), followed by resuspending in TPM buffer to an OD_600_ value of 40. To identify the effects of NaCl on the predation of strain MxC21, 2 μl or 200 μl of *E. coli* cells was placed in a TPM plate with the presence of NaCl and allowed to dry. Then, 2 μl of strain MxC21 was placed into the center or the edge of the *E. coli* prey colony. After incubation at 30°C for 2–3 days, spots were harvested with a loop and resuspended in 200 μl of TPM buffer. The number of surviving prey cells was counted by serial dilution on LB agar plates, on which strain EGB did not grow.

### 2.7. Whole-genome sequencing and functional annotation

The genome of strain MxC21 was sequenced using DNBSEQ and Nanopore platform at the Beijing Genomics Institute (BGI, Shenzhen, China). Short and long reads were obtained using the DNBSEQ (MGI) and the Nanopore platforms, respectively, of which the DNBSEQ (MGI) reads provided 1424.5-fold coverage and the Nanopore reads provided 300.2-fold coverage. The DNBSEQ sequencing generated 1,310 Mb of data, and the Nanopore platform sequencing produced 2.7 Gb of data. The data were subjected to quality control using SOAPnuke (v1.5.6; parameter, -l20-q40% -n10% -d) and Porechop (v0.2.4; default parameters) software. The program Canu (version 1.5 available at https://github.com/marbl/canu/releases; parameter, estn=24, npruseGrid=0corOvlMemory=4) was used for self-correction. Draft genomic unities were assembled using the Canu, a high-quality corrected circular consensus sequence subread set, and they are uncontested groups of fragments. To improve the accuracy of the genome sequence analysis, GATK (version 1.6–13 available at https://www.broadinstitute.org/gatk/; parameter, -cluster 2 -window 5 -stand_call_conf 50 -stand_emit_conf 10.0 -dcov 200 MQ0>=4) was used to make single-base corrections. The complete genome sequence of strain MxC21 was submitted to the NCBI GenBank with the accession number CP123278-CP123279. Gene prediction and functional annotation were performed by the online platform Rapid Annotation using Subsystem Technology (RAST) (Aziz et al., 2008; Sutton et al., 2019). To run the Cluster of Orthologous Group (COG) annotation, only the best blast hit was retained for each protein (Tatusov et al., 2000).

### 2.8. Quantitative PCR (RT-qPCR)

To evaluate the effects of salt on the growth of strain MxC21 in CTT medium, the gene expression levels of several key genes about the two-component system and phosphotransferase proteins and CRISPR-Cas were determined. The total RNA was extracted from the strains with different treatments using a HiScript III All-in-one RT SuperMix Perfect (Vazyme Biotech Co., Ltd, China). The total RNA was reverse-transcribed into cDNA using HiScript II QRT SuperMix for qPCR (Vazyme Biotech Co., Ltd, China) and used as a template for real-time quantitative reverse transcription-PCR (qRT-PCR) with Power SYBR Green PCR Master Mix (ABI). In total, 2 μl of the obtained cDNA solution was diluted 2-fold and mixed with specific primers (Supplementary Table S1) and AceQ^®^ Universal SYBR qPCR Master Mix (Vazyme Biotech Co., Ltd, China). The thermal cycling conditions for cDNA amplification involved an initial denaturation step at 95°C for 5 min, 40 × (95°C for 10 s and 60°C for 34 s), 95°C for 15 s, and a final melting curve (65–95°C, 0.5°C increment, for 5 s). All real-time quantitative PCR amplifications were run on the Applied Biosystems 7500 Real-Time PCR system. Gene expression values were calculated, relative to the housekeeping gene *rpoB*, by using the ^Δ^ΔCt method.

### 2.9. Comparative genomic analysis

To conduct a comparative genomic analysis of strain MxC21 with the other related myxobacterial species, the genome sequences of 10 species belonging to the genus *Myxococcus* were retrieved from the NCBI database (Supplementary Table S1). Predicted protein sequences for nine species were selected to perform the all-vs.-all blastp analysis with a cutoff value of 1 × 10^−5^. Then, blast scores were delivered to the MCL Markov clustering program with an inflation parameter of 2, resulting in rapid clustering of orthologous groups (Gibbons et al., 2015). Core proteome, dispensable proteome, and unique proteome were defined as proteins conserved in all the 9 *Myxococcus* genomes, 2–10 *Myxococcus* genomes, and only exist in the individual genome, respectively.

### 2.10. Phylogenetic analysis

Whole-genome phylogeny was performed to confirm the taxonomic classification. Based on the clustering results of orthologous proteins, we randomly selected 135 single-copy genes that conserved in all nine species (Edgar, 2004; Zhao et al., 2021). Alignments of the concatenated sequences of 135 single-copy genes were generated by the Muscle algorithm, and then, the phylogenetic tree was constructed by the neighbor joining algorithm with 1,000 bootstrap replicates in MEGA11 (Kumar et al., 2016). The synteny of *Myxococcus* sp. strain MxC21 genome and other bacterial genomes was determined using BLAST and Artemis Comparison Tool (ACT) (Carver et al., 2005). Otherwise, taxonomic annotations of the genomes were conducted with the toolkit GTDB-Tk v.1.4.0, according to the Genome Taxonomy Database (GTDB) (Parks et al., 2022). The average nucleotide identity (ANI) between genomes was counted using OrthoANI software (Yoon et al., 2017). The digital DNA–DNA hybridization (dDDH) values were calculated using the GGDC server with the recommended formula 2 (Meier-Kolthoff et al., 2013).

### 2.11. Statistical analysis

All experiments in the study were performed in triplicate with similar results, and the statistical significance was performed using Duncan's multiple comparisons test in SPSS 20.0 (IBM Inc., Chicago, IL, USA). The analytic results were expressed as mean values with standard deviations, and differences with *p* < 0.05 were considered statistically significant.

### 2.12. Data deposition

The whole-genome shotgun project was deposited in the NCBI/DDBJ/EMBL database under the accession number of CP123278-CP123279.

## 3. Results

### 3.1. Isolation of myxobacteria MxC21 with salt tolerance

**Figure 1:**
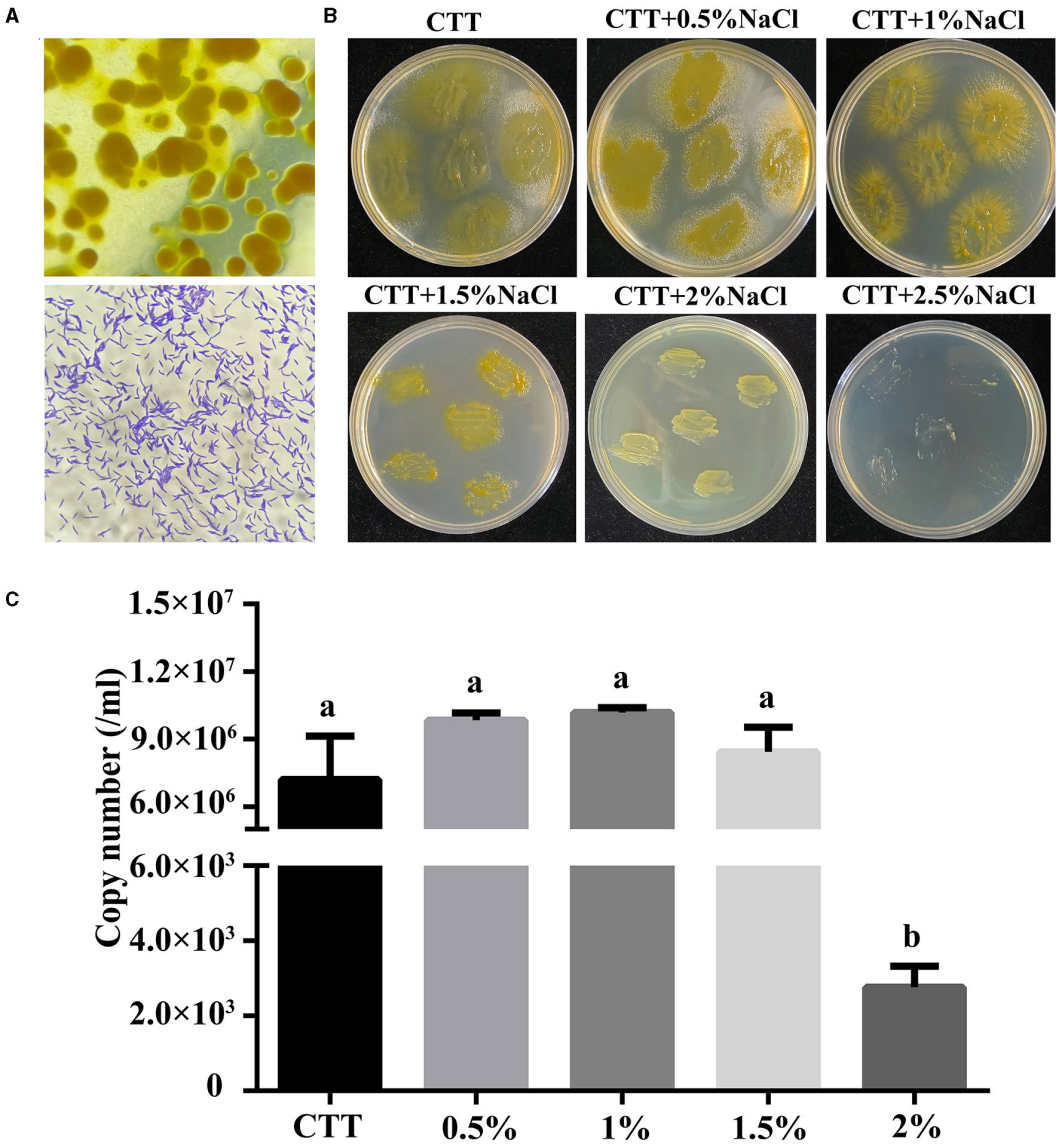
Salt-tolerant characteristics of strain MxC21. **(A)** Fruiting body formation and micrograph of strain MxC21 cells (1,000 ×); **(B)** salt-tolerant growth of strain MxC21 on CTT agar plates; **(C)** biomass analysis of strain MxC21 by qPCR. Error bars denote st. dev. (SD), and values with different letters indicate statistically significant differences (*p* < 0.05).

**Figure 2:**
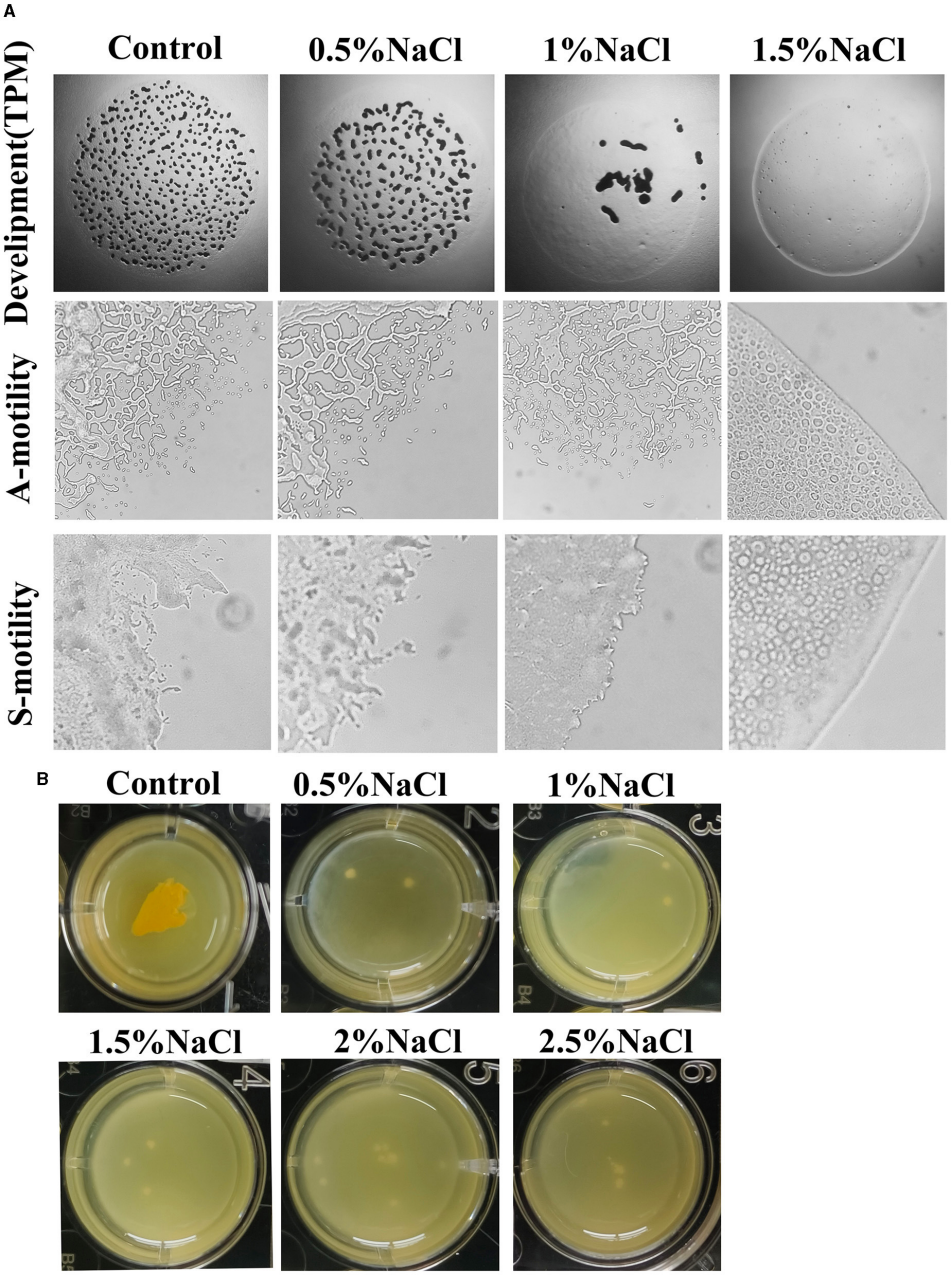
Effects of salt on the fruiting body formation and motility **(A)** and biofilm formation **(B)** of strain MxC21. The used concentration of NaCl is 0.5%, 1%, 1.5%, 2%, and 2.5% when necessary, and cultures without NaCl are used as control.

Using rabbit dung as the bait, 15 myxobacteria were isolated from forest soil in Xinjiang province *via* induction of fruiting body formation. The isolated strains were screened for their ability to grow in CTT medium with the presence of 1% NaCl (w/v). Among these isolates, strain MxC21 was selected as a promising salt-tolerant agent. Orange fruiting bodies were observed after induction by sterilized rabbit dung pellets for 3–4 days (Figure 1A). Moreover, strain MxC21 was a gram-negative bacterium with rod-shaped cell lacking flagella. To identify the salt-tolerant growth, strain MxC21 was cultured on the CTT agar plates containing NaCl with different concentrations (0–3%, w/v) (Figure 1B). The results showed that strain MxC21 tolerates NaCl concentrations as high as 1.5%, while growth inhibition of strain MxC21 was observed when the NaCl concentrations increased to 2% (Figure 2B). MxC21 cells clumped substantially in a liquid medium; we failed to determine the cell numbers by serial dilution. Hence, the biomass of strain MxC21 was qualified by qPCR. Strain MxC21 was able to grow in a liquid CTT medium containing 1.5% NaCl with maximal biomass, and limited growth with reduced biomass was identified with 2% NaCl (Figure 1C). This is consistent with the swarming growth of strain MxC21 on the CTT agar plate. These results indicate that wild myxobacterial isolate MxC21 from terrestrial soil can tolerate higher concentrations of NaCl with superior salt tolerance characteristics.

### 3.2. Social behavior and predation of MxC21 under salt condition

Social behavior of myxobacteria on solid surfaces is strongly affected by the salinity (Zhang et al., 2005), and the effects of NaCl on the development and motility of strain MxC21 were investigated. On the TPM agar plate containing 0.5% NaCl, typical development was observed after 3 days of inoculation. Whereas the number of fruiting bodies decreased, and consequently, few myxospores were produced with the concentration of NaCl increased to 1%. In total, 1.5% NaCl prevented the aggregation and sporulation of strain MxC21 (Figure 2A). Motility observation showed that 1% NaCl exhibits no effects on the A- and S-motility of strain MxC21, while obvious deficiency was observed for the motility of strain MxC21 when the concentration of NaCl increased to 1.5% (Figure 2A). These results are similar to the behavior of salt-tolerant strains HW-1 and DK1622 in response to different concentrations of NaCl or seawater (Pan et al., 2009).

The presence of biofilm surrounding bacterial cells confers protection against stress (Saidi et al., 2022). To determine the effects of salt on the biofilm formation of strain MxC21, the submerged cultures in 24-well plates were performed. As shown in Figure 2B, after incubation in MOPS buffer, biofilm formation was observed for strain MxC21 with the presence of 0.5–2.5% salt. This result indicates that strain MxC21 reserves the ability to produce extracellular matrix polysaccharide under salt conditions, which may be important for its salt tolerance and social behavior in a saline environment.

**Figure 3:**
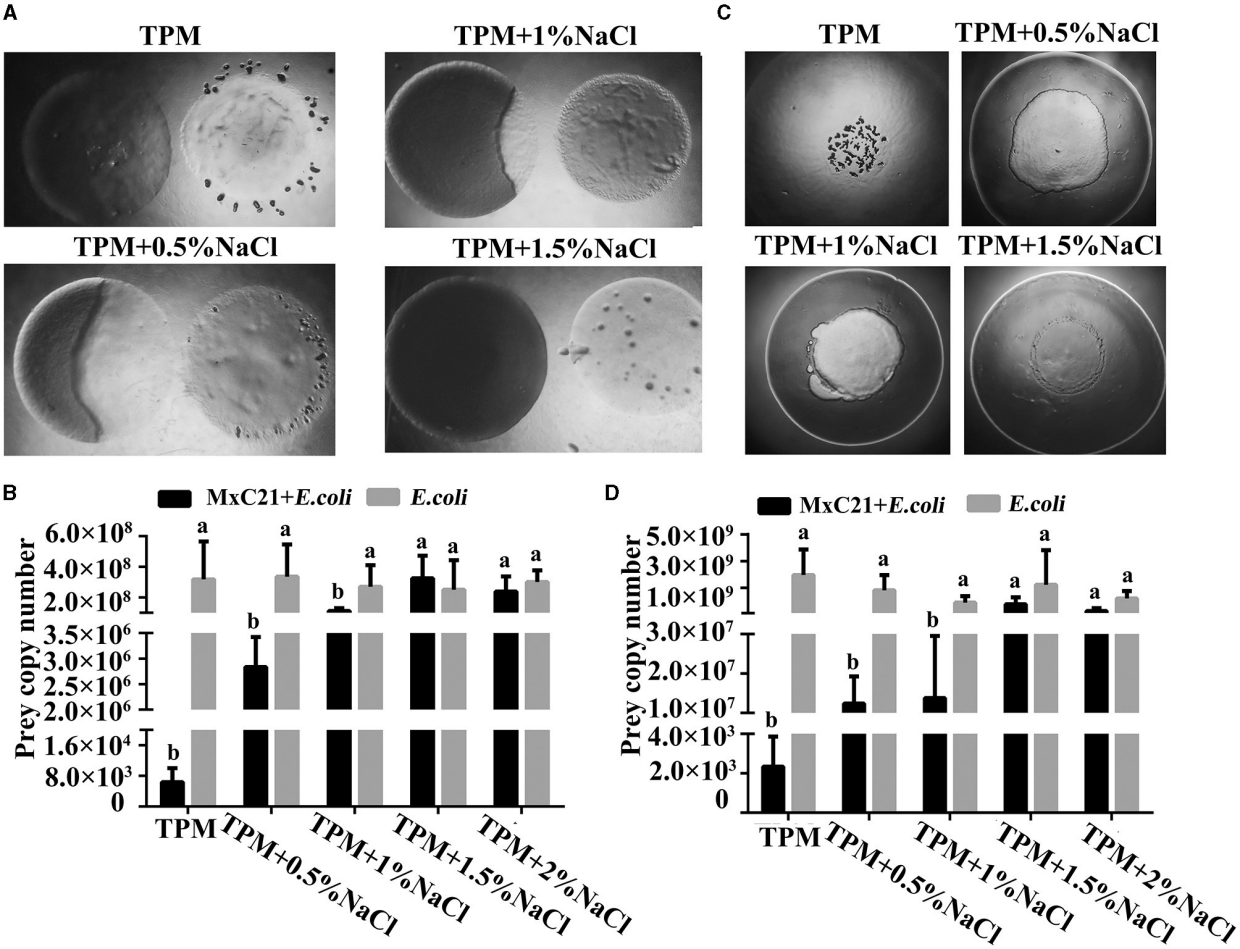
Effects of salt on the predation of strain MxC21. Strain MxC21 is cultured onto **(A)** or close to **(C)** the colony of *E. coli* on TPM plates with the presence of 0.5%, 1%, and 1.5% NaCl, and TPM plates without NaCl are used as control. **(B, D)** The viable cells of *E. coli* from the co-cultural assay **(A, C)** are also identified, respectively. The lowercases indicate the statistically significant differences (*p* < 0.05).

The predatory ability of strain MxC21 was also assessed under different salt conditions. Strain MxC21 was incubated onto (Figure 3A) or close to (Figure 3C) the colony of *E. coli* on TPM plates, and the swarming growth of strain MxC21 was obviously observed with prey *E. coli* as the sole nutrition with the presence of NaCl (0.5% and 1%). The predatory performance was assessed by counting the number of surviving cells of prey *E. coli* encountered by the expanding swarm of strain MxC21, which significantly decreased after the swarming predation when strain MxC21 was incubated onto (Figure 3B) and close to (Figure 3D) the colony of prey with the presence of NaCl (0.5 and 1%). These results indicate that strain MxC21 is able to feed on the bacteria prey under a saline environment. However, when the concentration of NaCl increased to 1.5%, strain MxC21 was unable to prey on bacteria (Figure 3), which was consistent with the motility deficiency (Figure 2). We deduced that strain MxC21 harboring the predatory ability is essential for its environmental adaptation.

### 3.3. Genome sequence assembly and general features

**Figure 4:**
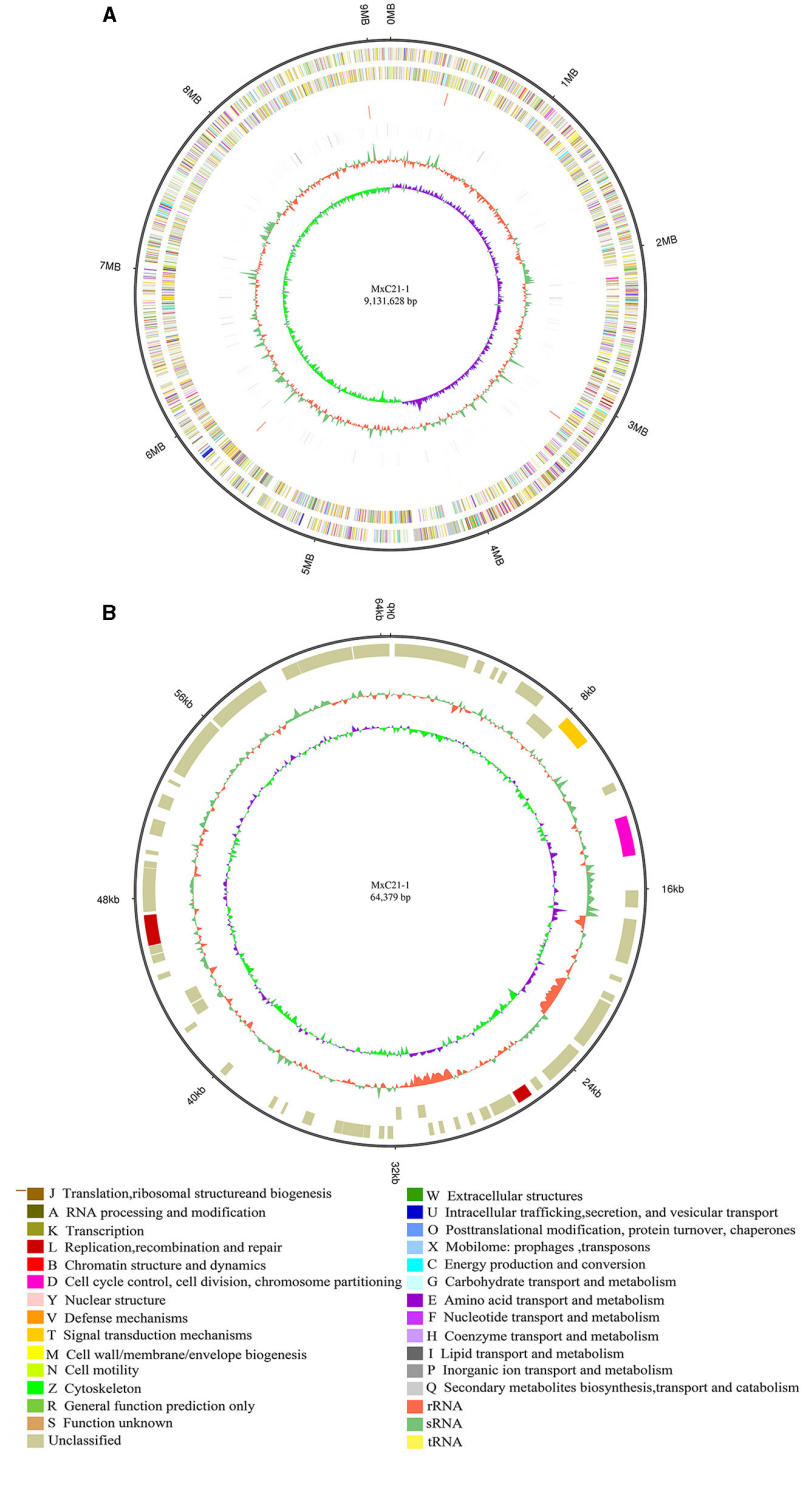
Circular representation of the complete genome **(A)** and plasmid **(B)** of strain MxC21. The outermost circle is the coordinate of the genome position. The corresponding circles from outside to the inside represent COG annotation gene distribution of the forwarding strand, COG annotation of the reverse strand, ncRNA distribution of the forwarding strand, and ncRNA distribution of the reverse strand, which depict the GC content and the GC skew, respectively.

The genome of strain MxC21 was sequenced using a hybrid strategy that combined sequences from Nanopore long reads and DNBSEQ short reads. The genome was assembled as a single circular chromosome of 9.41 Mbp with a high GC content of 69.13% (Figure 4A), and a circular plasmid of 64.3 kb with a GC content of 65.62% was also identified based on the genome sequence assembly (Figure 4B). Subsequently, RAST-based annotation has identified 9584 coding genes in the strain MxC21 genome, and 50.17% of the proteins were functionally annotated, while the remaining proteins (49.83%) were hypothetical proteins or proteins with unknown function. The identified indigenous plasmids in strain MxC21 were the second reported plasmid from myxobacteria; 68 coding genes in the strain MxC21 plasmid were identified, while 64 proteins were annotated as hypothetical proteins, and other 4 proteins were predicted as serine/threonine protein kinase, chromosome segregation protein, DNA repair protein, and DNA primase (Supplementary Table S3). The detailed functions and their potential relationship with the salt tolerance of strain MxC21 need further research. Otherwise, 31 ncRNAs were identified as clustered regularly interspaced short palindromic repeat (CRISPR) RNA direct repeat elements, which may provide acquired resistance against bacteriophages. These results indicated that the abundance of protein corresponding to signal transduction and regulation is possibly involved in the salt responses in myxobacteria with salt tolerance.

### 3.4. Taxonomic affiliation

**Figure 5:**
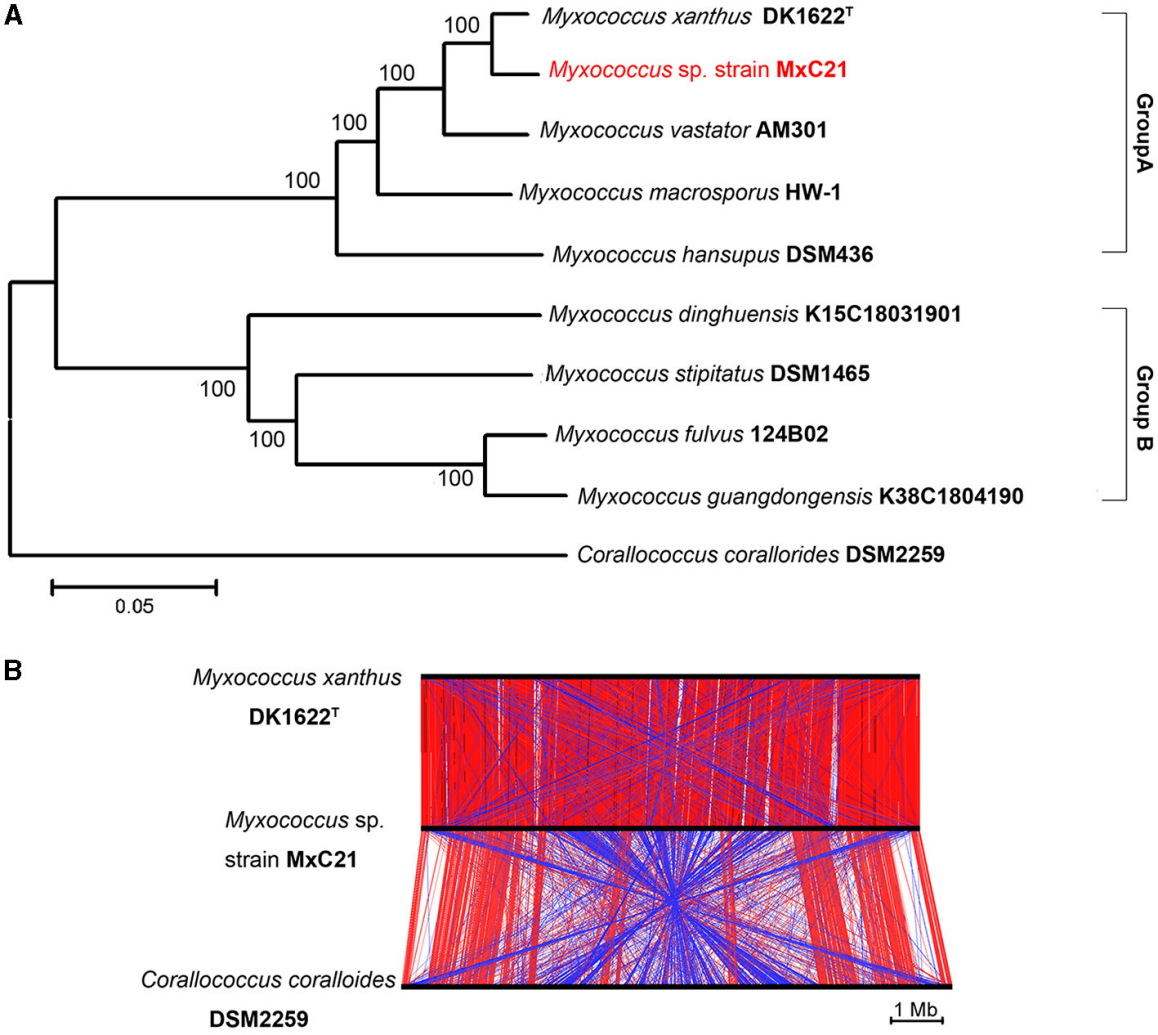
Phylogenetic relationships of strain MxC21 and related myxobacteria. **(A)** Construction of phylogenetic tree by the neighbor-joining algorithm with concatenated sequences of 135 single-copy genes. **(B)** Linear genomic comparison of strain MxC21, *M. xanthus* DK1622, and *C. coralloides* DSM 2259. The red lines represent matching regions, and the blue lines denote inverted matching regions.

Strain MxC21 was first identified as *Myxococcus* sp. and closely related to *M. xanthus* based on the phylogenetic analysis of the 16S rRNA gene. However, the polyphyletic nature of myxobacteria taxa makes its classification very difficult, according to the 16S rRNA gene analysis. To further establish the phylogenetic relationship of strain MxC21 with other related *Myxococcus* species, the phylogenetic tree was constructed using the concatenated sequences of 135 single-copy genes, which were conserved in all the analyzed genomes. As shown in Figure 5A, *Myxococcus* species grouped into two major clades, namely groups A and B. Strain MxC21 was located in the clade Group A clustering closely with *M. xanthus* DK1622. DK1622 is a model organism for studying bacterial social behaviors due to the feasible genetic manipulation. Hence, the alignment of three chromosomal sequences of strain MxC21, *M. xanthus* DK1622, and *C. coralloides* DSM 2259 was analyzed using ACT software. As shown in Figure 5B, strain MxC21 exhibited relatively high synteny with *M. xanthus* DK1622 but poor synteny with significant rearrangements from the genome comparison of strains MxC21 and DSM 2259. Meanwhile, the abundance of inverted matching regions was also observed between strains MxC21 and DK1622, although the two strains shared consistent phylogenetic lineage.

As a category of computational analysis, average nucleotide identity (ANI) is commonly used to define species boundaries, along with digital DNA–DNA hybridization (dDDH) (Kim et al., 2014). To further determine the evolutional lineage, the ANI and dDDH values between the above genomes were calculated. As shown in Table 1, all the ANI values ranged from 78.1% to 97% between the *Myxococcus* genomes. Meanwhile, strain MxC21 exhibited 97% ANI values and 73.5% dDDH values with *M. xanthus* DK1622 (Table 1), which was consistent with the phylogenetic relationship (Figure 5A). However, combining the linear genomic comparison and the low dDDH values indicates the possibility of strain MxC21 as a novel species within *Myxococcus* genus. Considering the high genome synteny of the genomes of strains MxC21 and DK1622, we provided a phylogenetically consistent and rank-normalized genome-based taxonomy by GTDB-Tk, according to the Genome Taxonomy Database (GTDB) based on approximately 120 marker genes. The result showed that strains MxC21 and DK1622 share similar evolutionary lines (Supplementary Figure S1). However, whether strain MxC21 belongs to members of *Myxococcus xanthus* needs more evidence.

**Table 1:** ANI and dDDH values for pairwise comparisons between *Myxococcus* species and *C. coralloides* DSM 2259.

**dDDH\ANI**	**MxC21**	**Mxan**	**Msti**	**Mful**	**Mhan**	**Mdin**	**Mgua**	**Mvas**	**Ccor**
MxC21	100.0	97.0	79.9	80.3	87.2	80.6	80.3	93.6	78.4
Mxan	73.5	100.0	79.8	80.3	87.1	80.5	80.2	93.5	78.2
Msti	22.5	22.3	100.0	83.6	79.7	81.9	83.4	80.2	78.1
Mful	22.9	22.9	26.5	100.0	80.1	82.7	94.1	80.7	78.6
Mhan	32.1	31.9	22.2	22.8	100.0	80.3	80.1	87.3	78.4
Mdin	23.3	23.2	24.6	25.4	23.1	100.0	82.3	80.9	78.9
Mgua	22.8	22.7	26.2	54.8	22.7	25.2	100.0	80.7	78.4
Mvas	52.8	52.8	23.0	23.5	32.9	23.8	23.5	100.0	79.0
Ccor	21.4	21.2	20.9	21.2	21.2	21.7	21.2	21.9	100.0

Average nucleotide identity (ANI) values (above) and digital DNA–DNA hybridization (dDDH) values (below) are present. Mxan, Myxococcus xanthus DK1622 (NC_008095.1); Msti, Myxococcus stipitatus DSM 14675 (NC_020126.1); Mful, Myxococcus fulvus 124B02 (CP006003); Mhan, Myxococcus hansupus strain DSM 436 (NZ_CP012109.1); Mdin, Myxococcus dinghuensis K15C18031901 (NZ_JAKCFB000000000.1); Mgua, Myxococcus guangdongensis K38C18041901 (NZ_JAJVKW000000000.1); Mvas, Myxococcus vastator AM301 (NZ_JAAIYB000000000.1); Ccor, Corallococcus coralloides DSM 2259 (NC_017030.1).

### 3.5. Genomic evidence for signal transduction and regulation

In response to high salinity stress, plants and microbes undergo a stress adaptive response and regulate a variety of genes whose products are involved in signal transduction, transcriptional regulation, and induction of stress tolerance by efficient protein folding involved in salt tolerance (Ayaz et al., 2022). Considering the salt tolerance characteristics of strain MxC21, we further analyzed the genes involved in signal transduction and regulation referring to the sequenced genome, as shown in Table 2. Strain MxC21 contains 285 two-component systems, 99 Ser/Thr kinases, 26 one-component systems, 235 transcription factors, and 121 histidine kinases, which mediate dominant response to a wide array of physiological changes, such as salinity stress (Pan et al., 2009). Otherwise, 26 one-component system proteins linking environmental signals to cellular responses were also identified. Otherwise, the genome of strain MxC21 contains 18 phosphotransferase proteins, higher than other *Myxococcus* species. Phosphotransferase proteins are group translocators that catalyze the uptake of hexoses or hexose derivatives and mediate cellular stress tolerance (Jamal et al., 2013). Considering the non-salt tolerance of the other *Myxococcus* species except for *M. xanthus* DK1622 and *M. fulvus*, the abundance of phosphotransferase proteins in strain MxC21 represents a potential function in environmental adoption.

**Table 2:** Variability in the numbers of regulatory genes among the *Myxococcus* species.

	**MxC21**	**Mxan**	**Msti**	**Mful**	**Mhan**	**Mdin**	**Mgua**	**Mvas**	**Ccor**
Ser/Thr kinases	99	99	111	100	94	113	108	113	107
Two-component system	285	282	296	322	301	315	329	294	305
Histidine kinase	121	141	153	168	159	167	175	149	162
Phosphotransferase protein	18	4	3	4	2	4	3	7	2
Response regulator	146	137	140	150	140	144	151	138	141
Transcription factor	235	270	372	400	295	378	404	256	351
One-component system	26	40	77	75	55	65	71	38	79
Response regulator	35	49	60	61	54	62	62	43	49
Sigma factors	51	56	67	72	48	63	67	52	56
Transcriptional regulators	123	125	168	192	138	188	204	123	167

### 3.6. Gene expression profiles of the signal response to the presence of salt

**Figure 6:**
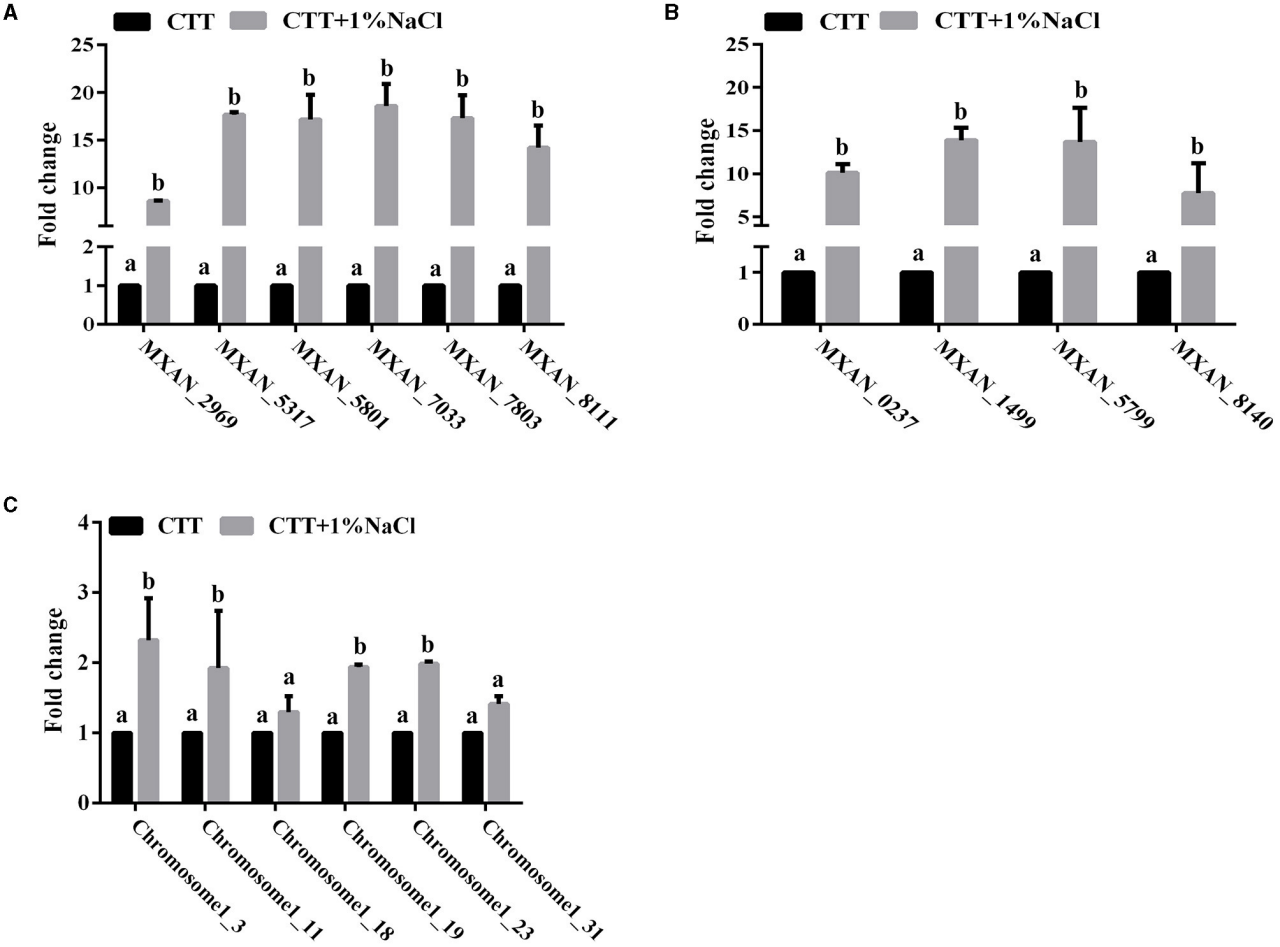
Expression profiles of the representative 6 TCS **(A)**, 4 phosphotransferase **(B)**, and 6 CRISPR-Cas **(C)** coding genes from strain MxC21 with the presence of 1% NaCl. The 6 TCS coding genes from strain MxC21 are MXAN_2969, MXAN_5317, MXAN_5801, MXAN_7033, MXAN_7803, and MXAN_8111; the 4 phosphotransferase coding genes from strain MxC21 are MXAN_0237, MXAN_1499, MXAN_5799, and MXAN_8140. The lowercases indicate the statistically significant differences (*p* < 0.05). The locations of the 6 CRISPR-Cas in chromosome of strain MxC21 are 1-3, 1-11, 1-18, 1-19, 1-23, and 1-31.

Adaptation of bacteria to the prevailing environmental stress is often mediated by regulatory proteins, which have essential functions in the sensing of external and self-generated signals in bacteria and the generation of appropriate output responses. The abundance of the two-component system and phosphotransferase proteins in the genome of strain MxC21 indicates the potential function in environmental adoption. To determine the response of these proteins toward salt stress, the expression levels of the representative 6 TCS and 4 phosphotransferase coding genes from strain MxC21 were studied with the presence of 1% NaCl. As shown in Figure 6, the presence of salt increased the gene transcript levels of all the 6 TCSs (Figure 6A) and 4 phosphotransferases (Figure 6B) of strain MxC21. These results indicate that these proteins corresponding to signal transduction and regulation are possibly involved in the salt responses of salt-tolerant myxobacteria. Otherwise, to identify whether CRISPR-Cas is active during growth under a saline environment, the expression levels of the representative 6 CRISPR-Cas genes in strain MxC21 were studied with the presence of 1% NaCl. As shown in Figure 6C, salt concentration promotes the gene transcript levels of all 4 CRISPR-Cas genes, indicating the active states of these genes from strain MxC21 in response to salt.

## 4. Discussion

Myxobacteria are ubiquitous micropredators that possess the capability to feed on a broad range of soil bacteria and fungi and oomycetes (Livingstone et al., 2017; Li et al., 2019; Zhang et al., 2023b), which are regarded as promising biocontrol agents for biocontrol of plant diseases (Ye et al., 2020; Luo et al., 2022). Myxobacteria have been considered as terrestrial bacteria, and abundant isolates of myxobacteria have been collected from soil environments. However, global geographic distribution showed that myxobacteria are one of the most diverse bacterial groups on earth with preferred appearances in non-saline soil and sediments, followed by saline environments (Wang et al., 2021b), whereas salt-tolerant myxobacteria have received less attention due to the limited resources and their adjustment to saline environments. Up to now, several myxobacteria with salt-tolerant properties have been isolated from seawater and sediments, and their predatory behavior under saline environment is largely unknown. In this study, we identified that a halotolerant *Myxococcus* sp. strain MxC21 isolated from forest soil displays typical social behaviors and predatory ability with the presence of 1% NaCl, and complex metabolic and signal pathways relating to salt tolerance were revealed by comparative genome and functional gene analysis. Salt-tolerant strain MxC21 represents fresh insight into the adaptive evolution of myxobacteria under terrestrial environment.

Salt-tolerant myxobacteria have been screened from marine enviroment, which mainly belong to the genus *Myxococcus* (Wang et al., 2007). Usually, terrestrial myxobacteria are salt sensitive and cannot grow at NaCl concentration higher than 1%, while genera of *Myxococcus, Cystobacter, Corallococcus, Sorangium, Nannocystis*, and *Polyangium* isolated from saline–alkaline soils also exhibit efficient growth with 1% NaCl (Zhang et al., 2013). *Myxococcus* sp. strain MxC21 isolated from forest soil exhibits favorable salt tolerance with 1.5% NaCl. Meantime, strain MxC21 still displays typical social behaviors such as motility and fruiting body formation with the presence of 1% NaCl. Commonly, colony expansion and development are restricted with increasing salt concentration, while the fruiting body structure of strain MxC21 was still completed under salt condition, but the number of fruiting bodies decreased. This is consistent with wild-type strain DK1622 (Pan et al., 2009). Previous research showed that horizontally transferred gene *Hdsp* is required for social behavior and halotolerance of *Myxococcus* strain during the adaptive evolution from terrestrial environment to liquid habitat (Pan et al., 2010). However, this gene is absent in genomes of strains MxC21 and DK1622, indicating that salt-tolerant soil myxobacteria present different lifestyles compared with marine isolates. Research studies of salt-tolerant myxobacteria indicate the potential adaptive evolution of myxobacteria from the terrestrial environment to liquid habitat, as social behaviors such as development and motility, which are important feathers for soil habitat, are lost in the liquid living environment. However, loss of social behaviors by *M. xanthu*s after 1,000 generations of evolution in a liquid habitat is also identified (Velicer et al., 1998), which raises the question of what is the role of salt during the evolution. The social behavior of strain MxC21 with the presence of 1% NaCl may inspire us to perform further study.

Genome sequencing of halotolerant *Myxococcus* is able to get a full picture of the evolutionary trial. In this study, we reported the complete genome sequence and functional annotation of strain MxC21. The phylogenetic tree is constructed based on the 135 single-copy genes, which indicate that strain MxC21 belongs to Group A of *Myxococcus* genus, closer to *M. xanthus* DK1622. However, linear genomic comparison and dDDH and ANI values revealed that strain MxC21 exhibits insufficient genomic similarity with the existing *Myxococcus* species, especially DK1622. Otherwise, HW-1 is a model myxobacterium for investigation of the social behavior from soil to oceanic condition (Pan et al., 2010; Li et al., 2011). Except for AM301 and DSM436, DK1622, HW-1, and MxC21 (Group A) all tolerate 1% NaCl during growth, indicating potential adaptation to salinity soil. Otherwise, biofilm formation is a common mechanism for surviving environmental stress (Kumar et al., 2016), and strain MxC21 reserves the abilities of predation and biofilm formation with the presence of 1% NaCl, which promotes the adaption to different habitats and thereby keeps population competition of myxobacteria.

Circular plasmid pMF1 from *M. fulvus* strain 124B02 is the first reported indigenous autonomously replicating plasmids from myxobacteria (Zhao et al., 2008), which raises the question of whether similar plasmids are present in myxobacteria. In strain MxC21, we found a circular plasmid of 64.3 kb according to genome sequence assembly, whereas strains MxC21 and 124B02 were distributed into different groups, indicating different functions of the plasmids. pMF1 from strain 124B02 has been identified to participate in natural genetic transformation (Zhao et al., 2008), and plasmid-mediated horizontal gene transfer (Li and Zhang, 2023) and pollutant degradation (Qin et al., 2015) were also identified. The appearance of indigenous plasmids in strain MxC21 may encourage us to consider their ecological function during the adaptive evolution of myxobacteria in a specific habitat.

Two-component systems and outer membrane proteins were involved in response to salt tolerance of *Myxococcus* cells (Pan et al., 2009), and adenylyl cyclase and Clp/HSP100 family of molecular chaperones were deduced to function in signal transduction during osmotic stress (Kimura et al., 2002; Pan et al., 2012). Genome analysis showed that the abundance of the two-component system and phosphotransferase proteins distributed in the genome of strain MxC21. In response to salt stress, expression levels of the representative genes encoding 6 TCSs and 4 phosphotransferases from strain MxC21 were significantly upregulated. The multifunctionality of these proteins involving signal transduction may enable myxobacteria to efficiently use cellular resources and acclimate to various environmental conditions.

## 5. Conclusion

In this study, a salt-tolerant myxobacterium *Myxococcus* sp. MxC21 was isolated from forest soil, which reserved predatory ability and social behaviors with the presence of 1% NaCl. Terrestrial myxobacteria are able to prey on bacteria under salt conditions, and diverse survival strategies enable myxobacteria to keep high environmental abundance. Phylogenomic analysis showed that strain MxC21 exhibits a phylogenetic relationship and diversity compared with other *Myxococcus* species. Strain MxC21 consists of a circular chromosome with a total length of 9.13 Mbp and a circular plasmid of 64.3 kb with abundant hypothetical proteins. Considering the wide distribution of proteins associated with signal transduction, transcriptional regulation, and protein folding, we deduce that strain MxC21 exhibits diverse niche adaptation toward different environments. The present study provides a preliminary inspiration for the social behaviors of salt-tolerant myxobacteria and the recognition of the adaptive evolution of myxobacteria. However, further investigations are required to investigate the underlying mechanisms.

## Data availability statement

The data presented in this study are deposited in the NCBI GenBank repository under accession number PRJNA956062.

## Author contributions

LL designed the experiments and performed the experiments. FX, PW, and LZ performed the experiments. JW, JZ, DM, XY, and YH analyzed the data. GH and ZC revised the manuscript. ZL wrote the manuscript and was responsible for the funding support. All authors read and approved the final manuscript.
