# Supplementary material for: Genome analysis of a plasmid-bearing myxobacterim Myxococcus sp. strain MxC21 with salt-tolerant property
Source: Front Microbiol. 2023 Sep 18;14:1250602. doi: 10.3389/fmicb.2023.1250602 (PMC10544341; doi:10.3389/fmicb.2023.1250602)
Supplement: Supplementary file 1 [file Data_Sheet_1.docx]

Supplementary Material

Article Title: Genome analysis of a plasmid-bearing myxobacterim *Myxococcus* sp. strain MxC21 with salt-tolerant property

Lin Liu^1^, Fengjuan Xu^2^, Jinhui Lei^1^, Peiwen Wang^1^, Lei Zhang^1^, Jihong Wang^1^, Jingya Zhao^1^, Dongmei Mao^2^, Xianfeng Ye^1^, Yan Huang^1^, Gang Hu^1, *^, Zhongli Cui^1^, Zhoukun Li^1, *^

# Supplementary Tables

## Supplementary Table 1

| Strain | NCBI Reference Sequence | Reference |
| --- | --- | --- |
| *Myxococcus sp. strain* MxC21 | CP123278 | This study |
| *Myxococcus xanthus* DK1622 | NC_008095.1 | This study |
| *Myxococcus stipitatus* DSM 14675 | NC_020126.1 | This study |
| *Myxococcus fulvus* 124B02 | CP006003.1 | This study |
| *Myxococcus hansupus* strain DSM 436 | NZ_CP012109.1 | This study |
| *Myxococcus dinghuensis* K15C18031901 | NZ_JAKCFB000000000.1 | This study |
| *Myxococcus guangdongensis* K38C18041901 | NZ_JAJVKW000000000.1 | This study |
| *Myxococcus vastator* AM301 | NZ_JAAIYB000000000.1 | This study |
| *Corallococcus coralloides* DSM 2259 | NC_017030.1 | This study |
| *Myxococcus macrosporus strain* HW-1 | CP002830.1 | This study |

## Supplementary Table 2

| Primer | Sequence (5’-3’) |
| --- | --- |
| MxC21GL002969_for | AGACGCAGAAGTCCGAAGCC |
| MxC21GL002969_rev | TTCACCGTGCCGATGATGT |
| MxC21GL005317_for | GCTCGTCTTCACCGACTCCC |
| MxC21GL005317_rev | ATCCAGGCGACCGATGTCC |
| MxC21GL005801_for | GGCAAGATCGTCGAGTTCATC |
| MxC21GL005801_rev | TCCTTCGTGGACTCCAGCAC |
| MxC21GL007033_for | CGGTGTCCGTGGCAGAA |
| MxC21GL007033_rev | GGCTCGCATTGTAGTAGCG |
| MxC21GL007803_for | CGGCGAGAAGACGCAGGAA |
| MxC21GL007803_rev | GCTCACGACGGAGGTGGACT |
| MxC21GL008111_for | TTCGGACTGTCGCTACTGATG |
| MxC21GL008111_rev | CGGATGATGCGGAAGTTCTC |
| Rpob_for | CAAGGACCAGAACGACGAGA |
| Rpob_rev | CCAGGTTGCGGAGGATGT |
| MxC21GL000237_for | ATCGCGCACGAAATCAA |
| MxC21GL000237_rev | TTCACAGGCCTCTTGC |
| MxC21GL001499_for | TCGCGCTGATTACCGC |
| MxC21GL001499_rev | ACGGTGGCGTAGAGCA |
| MxC21GL005799_for | TCAGGCAGTTCCGGGA |
| MxC21GL005799_rev | ATCTCGTGCACCAGCA |
| MxC21GL008140_for | ACCTCGCGGACTTCC |
| MxC21GL008140_rev | TTGATGGTGTGCACGG |
| Chromosome1_3_for | GAGGCTGATTTTCGGGCTCCGG |
| Chromosome1_3_rev | GGAGCCCGAGAATCAGCCTCCGGA |
| Chromosome1_11_for | GGACCTCACGGGACGATGC |
| Chromosome1_11_rev | CGTCATTCCTGGCGGCATC |
| Chromosome1_18_for | CCGAGCCGTCACATCCTG |
| Chromosome1_18_rev | CAGCGTCCCTTGAGACGCT |
| Chromosome1_19_for | CAATGAGGGGACCACCGAGG |
| Chromosome1_19_rev | TCATTGGCCGGTTTTGCTC |
| Chromosome1_23_for | TGTTGAGGCAGGGTTGGC |
| Chromosome1_23_rev | AGCGGCGCAGCAGGAA |
| Chromosome1_30_for | CGGCGGGCGTCTCCAGCCACAC |
| Chromosome1_30_rev | GTGTGGCTGGAGACGCCCGCCG |
| Chromosome1_31_for | ACCCGAGCACAGCAGGACA |
| Chromosome1_31_rev | TGCGAGCCAAGTGGAGCAG |

Primer used for RT-qPCR of two-component system, phosphotransferase proteins and CRISPR-Cas genes

## Supplementary Table 3

| ID | Position (bp) | Size of product (amino acids) | COG:Anno |
| --- | --- | --- | --- |
| MxC21-1GL008768 | 181-3297 | 1039 | NA |
| Prophage:pp18 | 181-54344 | 21388 | NA |
| MxC21-1_TR688 | 3356-3407 | 17 | NA |
| MxC21-1_TR689 | 3387-3512 | 42 | NA |
| MxC21-1GL008769 | 3696-4007 | 104 | NA |
| MxC21-1GL008770 | 4448-4639 | 64 | NA |
| MxC21-1GL008771 | 4811-5041 | 77 | NA |
| MxC21-1GL008772 | 5713-6843 | 377 | NA |
| MxC21-1GL008773 | 6935-9071 | 379 | NA |
| MxC21-1GL008774 | 8126-9430 | 435 | Serine/threonine protein kinase HipA, toxin component of the HipAB toxin-antitoxin module |
| MxC21-1_TR690 | 10101-10164 | 21 | NA |
| MxC21-1_TR691 | 10146-10281 | 45 | NA |
| MxC21-1_TR692 | 11024-11317 | 98 | NA |
| MxC21-1GL008775 | 11436-11801 | 122 | NA |
| MxC21-1GL008776 | 12914-14590 | 559 | Chromosome segregation protein Spo0J, contains ParB-like nuclease domain |
| MxC21-1GL008777 | 16056-16778 | 241 | NA |
| MxC21-1_TR693 | 16770-16935 | 55 | NA |
| MxC21-1GL008778 | 17280-19157 | 626 | NA |
| MxC21-1_TR694 | 17448-17666 | 73 | NA |
| MxC21-1_TR695 | 17448-17941 | 164 | NA |
| MxC21-1GL008779 | 19770-19973 | 68 | NA |
| MxC21-1GL008780 | 20564-20863 | 100 | NA |
| MxC21-1_sRNA_000005 | 20912-20952 | 13 | NA |
| MxC21-1GL008781 | 20964-22466 | 501 | NA |
| MxC21-1_TR696 | 21438-22033 | 198 | NA |
| MxC21-1GL008782 | 22430-23044 | 205 | NA |
| MxC21-1GL008783 | 23326-25023 | 566 | NA |
| MxC21-1GL008784 | 25384-25695 | 104 | NA |
| MxC21-1GL008785 | 25968-26609 | 214 | DNA repair protein RadC, contains a helix-hairpin-helix DNA-binding motif |
| MxC21-1GL008786 | 26748-27743 | 332 | NA |
| MxC21-1GL008787 | 27886-28239 | 118 | NA |
| MxC21-1GL008788 | 28586-28813 | 76 | NA |
| MxC21-1GL008789 | 29232-29426 | 65 | NA |
| MxC21-1_TR697 | 29635-30586 | 317 | NA |
| MxC21-1GL008790 | 29908-30102 | 65 | NA |
| MxC21-1GL008791 | 30361-30555 | 65 | NA |
| MxC21-1GL008792 | 30573-30917 | 115 | NA |
| MxC21-1GL008793 | 31692-31940 | 83 | NA |
| MxC21-1GL008794 | 32079-32318 | 80 | NA |
| MxC21-1GL008795 | 32454-32681 | 76 | NA |
| MxC21-1GL008796 | 33056-33328 | 91 | NA |
| MxC21-1GL008797 | 33339-34229 | 297 | NA |
| MxC21-1GL008798 | 34241-34573 | 111 | NA |
| MxC21-1GL008799 | 35541-35939 | 133 | NA |
| MxC21-1GL008800 | 36788-36907 | 40 | NA |
| MxC21-1GL008801 | 37279-37476 | 66 | NA |
| MxC21-1GL008802 | 39674-39958 | 95 | NA |
| MxC21-1_TR698 | 40751-40776 | 8 | NA |
| MxC21-1GL008803 | 42053-42259 | 69 | NA |
| MxC21-1GL008804 | 42445-42972 | 176 | NA |
| MxC21-1GL008805 | 43035-43655 | 207 | NA |
| MxC21-1GL008806 | 44524-44745 | 74 | NA |
| MxC21-1GL008807 | 45254-45589 | 112 | NA |
| MxC21-1GL008808 | 45633-45980 | 116 | NA |
| MxC21-1GL008809 | 46006-47295 | 430 | DNA primase (bacterial type) |
| MxC21-1GL008810 | 47427-49271 | 615 | NA |
| MxC21-1_TR699 | 47905-47944 | 13 | NA |
| MxC21-1GL008811 | 49292-49561 | 90 | NA |
| MxC21-1GL008812 | 49857-50024 | 56 | NA |
| MxC21-1GL008813 | 50689-51351 | 221 | NA |
| MxC21-1GL008814 | 51874-51437 | 188 | NA |
| MxC21-1GL008815 | 52974-53117 | 48 | NA |
| MxC21-1GL008816 | 53459-56041 | 861 | NA |
| MxC21-1GL008817 | 56184-56984 | 267 | NA |
| MxC21-1GL008818 | 56984-58723 | 580 | NA |
| MxC21-1GL008819 | 59718-60413 | 232 | NA |
| MxC21-1GL008820 | 60427-62760 | 778 | NA |
| MxC21-1GL008821 | 62815-64344 | 510 | NA |

Predicted ORFs in the complete sequence of MxC21 plasmid, NA indicates no annotation with hypothetical protein.

## Supplementary Figure S1


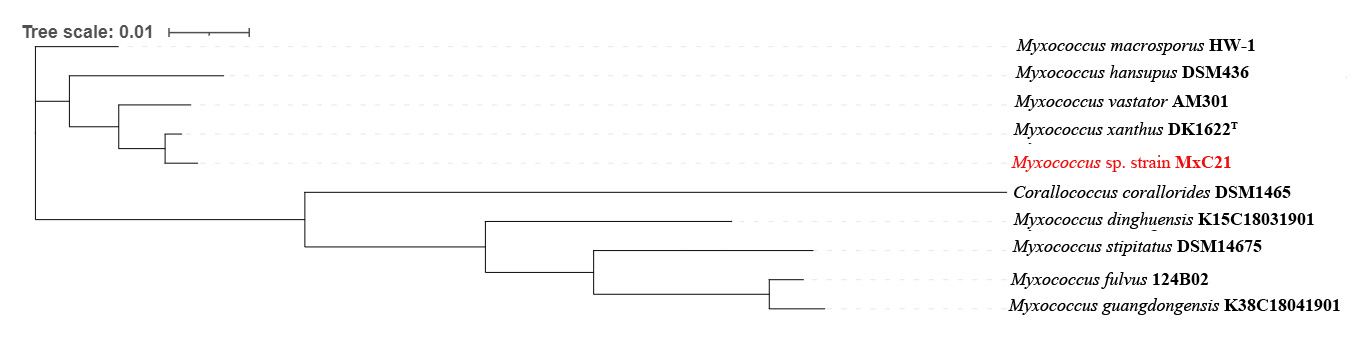


Figure S1 Construction of taxonomic annotations of the genomes of *Myxococcus* sp. strain MxC21were and other strain with the toolkit GTDB-Tk
